# Power-Assisted Liposuction of the Superomedial Pedicle in Primary Wise-Pattern Reduction Mammoplasties

**DOI:** 10.3390/jcm14134475

**Published:** 2025-06-24

**Authors:** Ines Ana Ederer, Shadi Najaf Zadeh, Jonas Walber, Florian Johannes Jung, Abdul Rahman Jandali, Alberto Franchi

**Affiliations:** 1 Department of Plastic and Aesthetic, Reconstructive and Hand Surgery, AGAPLESION Markus Hospital, 60431 Frankfurt am Main, Germany; 2 Department for Oral, Cranio-Maxillofacial and Facial Plastic Surgery, University Hospital Frankfurt, Goethe University, 60590 Frankfurt am Main, Germany; 3 Breast Center Zurich, 8008 Zurich, Switzerland; shadi.najafzadeh@brust-zentrum.ch; 4 Department of Hand and Plastic Surgery, Cantonal Hospital of Winterthur, 8401 Winterthur, Switzerland; jonas.walber@ksw.ch (J.W.); florian.jung@ksw.ch (F.J.J.); abdulrahman.jandali@ksw.ch (A.R.J.); albertofranchimd@gmail.com (A.F.)

**Keywords:** breast reduction, liposuction, superomedial pedicle, macromastia

## Abstract

**Background:** Superomedial pedicle breast reduction is a widely performed procedure in plastic surgery. However, in cases of massive ptosis and excessively large breasts, achieving adequate pedicle reduction can be challenging. Direct excision of the tissue bulk may compromise blood supply while insufficient reduction can hinder proper pedicle positioning or result in strangulation when forcefully placed in the keyhole area. This study investigates the application of power-assisted liposuction (PAL) to the superomedial pedicle, aiming to achieve volume reduction while preserving its vascular integrity. **Methods:** Patients who underwent reduction mammaplasty with concomitant PAL were retrospectively reviewed. Parenchymal resection was performed first, followed by PAL, which was selectively applied to the pedicle. Eligibility for liposuction was made intraoperatively based on breast morphology and the ease of pedicle insetting. **Results**: The mean lipoaspirate per breast was 243.0 mL (SD 131.3) following a mean resection weight of 1261.7 g (SD 356.9). In 76.7% of cases, more than 150 mL was aspirated. The smallest volume per breast was 50 mL, while the highest reached 500 mL. A strong correlation was observed between the aspirated volume and resection weight. The overall complication rate was 3.3%, with one patient requiring hematoma evacuation. No cases of NAC necrosis occurred. All patients reported satisfactory breast shape and size. **Conclusions**: Power-assisted liposuction of the superomedial pedicle is a reliable and efficient technique for reshaping and reducing the pedicle while maintaining a low risk of complications.

## 1. Introduction

Breast reduction surgery, or reduction mammoplasty, is one of the most frequently requested procedures in the female population. According to the global survey by the International Society of Aesthetic Plastic Surgery (ISAPS), over 686,000 procedures were performed worldwide in 2023 [1]. This number underscores the procedure’s reputation as an effective, reliable, and well-accepted technique for reducing excessive breast volume and alleviating symptoms associated with morbid macromastia.

Given the considerable variability in breast size, degree of ptosis, skin quality, and tissue consistency among patients, breast reduction can be performed by a variety of surgical techniques, including different pedicle designs, incision patterns, and liposuction [2,3,4,5,6,7,8,9,10,11]. Although each technique offers certain advantages and disadvantages, all of them share the common goal of achieving an aesthetically pleasing breast size and shape while preserving the integrity of the nipple–areolar complex (NAC). In line with this objective, pedicled breast reduction surgery is considered superior to free nipple grafting, particularly in terms of aesthetic outcomes and functional preservation [12,13,14]. Thus, it should be the primary option to treat gigantomastia, as supported by a recent meta-analysis [15].

However, achieving optimal results with pedicled techniques in cases of very large breasts with severe ptosis can be challenging. While excessive thinning of the skin flaps or the NAC-carrying pedicle can lead to tissue necrosis, insufficient volumetric reduction may also result in considerable postoperative complications. As such, an overly bulky pedicle can create disproportionate volume distribution, compromise accurate positioning of the NAC on the breast mound, and restrict the overall degree of breast reduction. In addition, forcing an inadequately reduced pedicle into the keyhole area may result in kinking or strangulation, thereby increasing the risk of tissue necrosis and adversely influencing wound healing and aesthetic results.

To overcome these limitations and mitigate the need for free nipple grafting, a hybrid technique combining breast liposuction with parenchymal resection can be employed. While this approach is not new, with liposuction being traditionally used to target areas such as the lower and lateral poles or the pre-axillary region, some authors have extended its application to the pedicle [10,16,17,18,19,20,21]. In 2015, Abboud et al. described their experience with power-assisted liposuction (PAL) for laterally pedicled breast reductions [20]. According to their approach, liposuction is performed before glandular resection, aiming to minimize skin undermining and parenchymal resection while preserving maximal blood supply. In contrast, a recent single-center study involving 189 patients utilized PAL to reduce the superior pedicle following its dissection and conventional parenchymal resection of the lower and lateral quadrants. However, the average volume of lipoaspirate was low—averaging 8 mL per breast (range 5–19 mL)—raising questions about the clinical benefit of the adjunctive procedure in this study [16].

In our practice, we have been using PAL to selectively target the pedicle since 2021. As our aspirated volumes notably exceed the values mentioned above, we prefer to use a superomedial pedicle, which ensures a more robust blood supply [22]. In contrast to others, liposuction is performed after parenchymal resection in our protocol. The primary goal of incorporating concomitant liposuction into breast reduction is to substantially reduce the volume of the pedicle, increase its pliability, and facilitate its positioning [20]. Ultimately, this minimizes the need for aggressive thinning and undermining, thereby preserving the vascular and neuronal integrity of the NAC and surrounding skin envelope. In this study, we want to share our experience with this hybrid method and emphasis the importance of individualized treatment plans for improving patient outcomes.

## 2. Materials and Methods

In this retrospective study, the charts of all patients who underwent non-oncologic breast reduction from 2021 were reviewed. The indication for breast reduction surgery was based on symptoms such as skin irritations in the inframammary fold, posture problems due to the excessive weight of the breasts, presence of shoulder ruts, and chronic neck, shoulder, or back pain; thus, all surgical procedures were covered by the statutory health insurance companies. Unilateral procedures and patients with secondary breast reductions or prior breast surgeries were excluded from this study. Each patient underwent careful clinical examination and radiographic assessment by ultrasound and mammography (for patients older than 40 years) before the operation. This screening allowed for the exclusion of neoplastic breast disease.

### 2.1. Surgical Technique

All procedures were carried out by the senior author (A.F.) and performed under general anesthesia. Since all patients sought a significant reduction in breast volume, a Wise-pattern incision was used. The pedicle was positioned superomedially in all patients.

**Figure 1:**
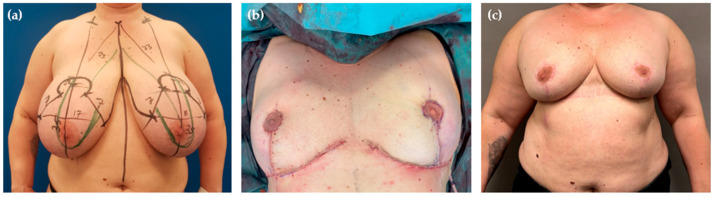
Power-assisted liposuction of the superomedial pedicle in a 41-year-old patient. (**a**) Preoperative markings, with PAL performed in the pedicle area (indicated by green lines). Black lines represent standard incision markings for Wise-pattern breast reduction [23,24]. The numbers indicate specific landmarks for surgical incision points (e.g., “23”cm marks the new superior edge of the NAC from the suprasternal notch, and “7”cm the length of vertical limbs). (**b**) Intraoperative view following resection of 1400 g on each side and liposuction of 300 mL per breast. (**c**) Postoperative result 6 months after surgery.

Preoperative markings were carried out with the patients in standing position. The chest midline, breast meridians, and the inframammary fold were marked on both sides. The new position of the NAC was located 8–10 cm below the upper breast border, which usually lies a few centimeters cranial to the anterior projection of the inframammary fold (IMF), also known as Pitanguy’s point. Then, standard markings for the Wise-pattern incision were carried out [23,24]. The areolar diameter was set at 4 cm while the length of the vertical limbs ranged between 6–7 cm. The superomedial pedicle was marked from the apex of the keyhole pattern, passing around the NAC in a slightly U-shaped curve, to the inferior third of the medial limb of the vertical incision (see Figure 1a). A 38 mm cookie cutter was used to mark the new desired NAC circumference. The pedicle and the NAC were then incised, and the pedicle was deepithelialized and developed by dissecting down to the pectoralis fascia. Parenchymal resection of the remaining breast tissue within the Wise pattern (lateral and lower quadrants) was performed according to preoperative markings. After achieving hemostasis, the NAC was rotated into its new position. The ease of insetting and the pliability of the tissue were critically evaluated during this simulation. If excessive tension, persistent tissue bulk, or disproportionate tissue mass in the upper pole was present, the decision for additional liposuction of the superomedial pedicle was made instead of continuing with aggressive resection, or wide undermining of the breast tissue or converting to free nipple grafting.

After infiltrating with tumescent solution (1000 mL isotonic saline, 500 mg of 1% lidocaine, and 3 mL epinephrine 1/1000), PAL was performed using 5.0 mm blunt-tipped cannulas. Liposuction was confined to the pedicle rather than the remaining breast tissue. The cannula was inserted at the caudal border of the pedicle. For proper fat harvest, the pedicle was held in the surgeon’s non-dominant hand while liposuction was performed with the dominant hand. Liposuction was accomplished in several planes parallel to the skin. Intraoperative simulation of NAC rotation was repeated, and liposuction continued until the desired amount of reduction was achieved. The aim was to effortlessly position the pedicle within the keyhole area and to facilitate tension-free skin closure.

The quantification of lipoaspirate was performed visually after the total aspirate had settled into separate layers. The adipose volume of the lipoaspirate was calculated by subtracting the supernatant fluid (oil layer on the top) and infranatant fluid (aqueous layer on the bottom) from the total aspirate volume.

Further steps of the operation were performed in standard procedure: after the NAC was rotated in the center of the keyhole, the medial and lateral pillars were brought together, and wound closure was obtained in a multilayered fashion. Drains were routinely placed on each side and removed once drainage volume decreased to below 30 mL within 24 h. We did not perform fascial suturing to the pedicle and did not use mesh techniques for internal breast support. At the conclusion of the operation, a tight-fitting compression bra was applied, which was recommended to be worn for a total of six weeks postoperatively. A video summarizing the operative technique can be assessed in the Supplementary Materials.

The primary endpoint of the study was defined as necrosis of the NAC or the pedicle. Secondary endpoints were further postoperative complications such as hematoma, seroma, wound dehiscence, or infection.

### 2.2. Statistical Analysis

The results are displayed as means and standard deviation (SD) or median and interquartile range (IQR) in case of non-normally distributed data. Categorial variables are reported as frequencies and proportions. The association between resection weight and liposuction volume was assessed using linear regression analysis. Statistical analysis was performed using IBM SPSS Statistics 26. A *p*-value less than 0.05 was considered significant.

## 3. Results

From 2021 onwards, we performed 30 PAL-assisted superomedial pedicle reduction mammoplasties. All procedures were performed bilaterally. The mean patient age was 42.1 years (SD 15.9). The mean BMI was 30.9 (SD 4.9). Except for two patients, all others were regarded as overweight (13.3%) or obese (73.3%). Three patients (20.0%) were active smokers at the time of surgery. None of the patients had a history of bleeding complications, coagulation disorders, or the use of antithrombotic drugs.

**Figure 2:**
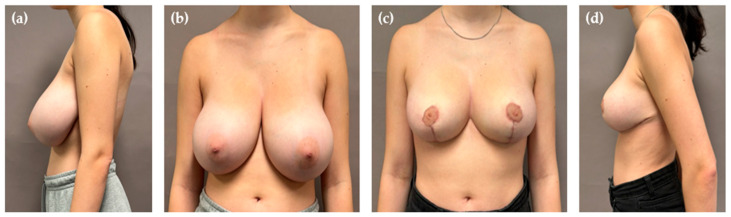
Preoperative view of 20-year-old women (**a**,**b**). Postoperative view (8 months) following PAL-assisted reduction mammoplasty (**c**,**d**). Liposuction volume was 50 mL per side following resection of 650 g on the right and 680 g on the left side.

The mean preoperative sternal notch-to-nipple distance was 36.8 cm (SD 5.3) which was corrected by a median NAC elevation of 12 cm (IQR 5). The decision to perform concomitant liposuction was made intraoperatively following conventional resection of the lateral and lower quadrants. When difficulties with pedicle insetting were encountered due to its persisting volume, liposuction was applied until the desired reduction and mobility were achieved. The mean lipoaspirate per breast was 243.0 mL (SD 131.3) following a mean resection of 1261.7 g (SD 356.9) per breast. In 23 out of 30 breasts (76.7%) the lipoaspirate was more than 150 mL. The smallest volume of lipoaspirate per breast was 50 mL, obtained from a 20-year-old patient who underwent a resection of 650 g on the right and 680 g on the left side, respectively (Figure 2). In contrast, the largest volume of lipoaspirate was 500 mL on the right and 450 mL on the left side. This was obtained in a patient in whom 1750 g was resected from the right and 1450 g from the left breast.

**Figure 3:**
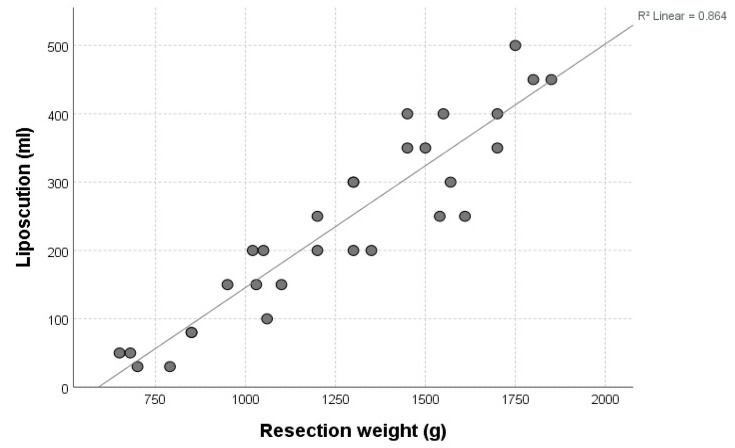
Positive correlation between resection weight (*x*-axis, grams) and liposuction volume (*y*-axis, milliliters).

In general, there was a strong positive correlation between the amount of lipoaspirate and resection weight, as depicted by Figure 3 (R^2^ 0.864, *p* < 0.001). The mean operative time for the entire procedure was 132 min (SD 24.7). All mammary samples obtained by standard resection were sent for histopathological examination. No pathologic findings were reported. A detailed summary of key demographic and intraoperative characteristics of all cases is given by Table 1.

The overall complication rate was low, with one major complication in the entire cohort (3.3%). The need for operative revision was related to the evacuation of unilateral hematoma, which originated outside the area of liposuction, on postoperative day 1. There were no vascular perfusion problems, such as transient venous congestion or arterial malperfusion of the NAC, in any case. Thus, no partial or complete necrosis of the NAC occurred. Two patients developed an oil cyst on the lateral aspect of the breast, both of which were successfully treated via local puncture and showed no recurrence. Otherwise, there were no perfusion problems of the parenchymal tissue that could be attributed to the adjunctive liposuction. None of the patients presented with postoperative seroma or infection during a mean follow-up of 6.2 months (SD 2.3, range 4–24 months).

**Figure 4:**
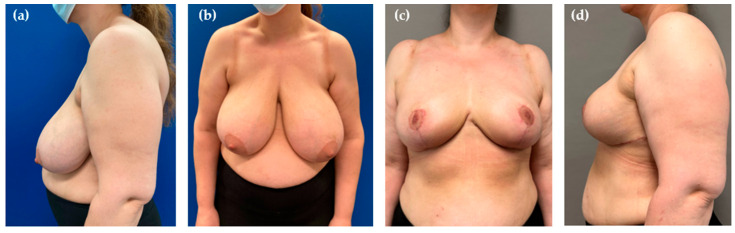
Preoperative view of a 43-year-old patient with massive ptosis and posture problems due to the excessive weight of the breasts (**a**,**b**). Results 6 months after 1200 g of resection and 200 mL of liposuction per side (**c**,**d**).

Patient satisfaction was universally high, with all agreeing to undergo the same procedure again. All women reported being very satisfied with their breast shape and size. There were no skin irregularities in the upper pole attributable to the performed liposuction (Figure 4). No revision surgeries were undertaken for aesthetic reasons such as contouring, re-reduction, or scar correction.

## 4. Discussion

As Orlando and Guthrie noted in 1975, “*A principal concern in both reduction mammaplasty and dermal mastopexy has been to find a safe, suitable, and technically easy method of transposing the nipple to its new bed while maintaining viability and normal sensation and obtaining a cosmetically acceptable result*” [7]. Their initial description of the superomedial pedicle set the foundation for an approach that prioritizes vascular safety, nipple viability, and optimal breast shape. Over time, breast reduction has continuously evolved, with Madeleine Lejour being another pioneer that shaped current techniques, particularly regarding the use of vertical reduction. In 1990, she stated that “*in the future the combination of fat aspiration and surgical excision will likely become the best way to reduce large breasts*” [10]. Her innovative approach involved liposuction as a complementary procedure before surgical resection to facilitate modeling of the breast [10]. Although she initially asserted that liposuction can be performed in all parts, she later recommended sparing the areolar part [5,10]. This shift in approach was further adopted by other surgeons, who predominantly employed liposuction to reduce lateral breast fullness or axillary excess [17,18,19,21].

Our technique differs in the implementation of liposuction from these studies. We first perform parenchymal resection, followed by liposuction of the pedicle, from its tip to the parasternal attachments, including the retroareolar part. This approach is based on the premise that liposuction preserves the vascular and neuronal network, a concept that is supported by findings in other surgical applications [25,26,27,28,29,30]. Rather than compromising perfusion, liposuction maintains the pedicle’s integrity while reducing its volume due to selectively removing fat by aspiration with blunt cannulas [20,31]. In contrast to Abboud et al., who utilized PAL for laterally pedicled breast reductions, our technique did not focus on reducing the overall amount of parenchymal resection through liposuction [20]. We selectively targeted the pedicle to facilitate its insetting and reduce its volume, addressing only this area as needed.

Unlike other colleagues who shared their experience with “liporeduction of the breast”—a technique which involves liposuction of the entire tissue followed by mastopexy—we do not consider breast liposuction to be “fast and easy” [31]. First, the additional installation of the equipment and its use need to be taken into account. Although we have not specifically measured the extra time, about 15–20 min should be allocated for performing PAL following resection. Secondly, employing liposuction on one side aggravates the performance of other relevant operative steps on the other side due to movements on the operating table. This means that liposuction is either performed on both sides simultaneously or it is performed sequentially, one side at a time. In addition, liposuction can sometimes be physically demanding, requiring considerable effort to aspirate the desired volume for pedicle reduction, even in well-selected patients [5]. This is related to the fact that deep to the areola the breast tissue is more concentrated than in other parts [10]. Thus, we underline that complimentary PAL of the pedicle provides no universally applicable solution for all patients or rather in all settings. In our hands, only a small percentage of patients is the right candidate for the hybrid method, which explains the small number presented in this study. In most cases, there is no need for additional liposuction in superomedial breast reductions. Even when the pedicle is very long, it can often be sufficiently debulked to fit within the proposed keyhole area. However, we find this technique particularly beneficial in females with very large, ptotic breasts that still exhibit a considerable amount of projection (Figure 1, Figure 2 and Figure 4). In such cases, the pedicle may retain excessive fullness even after thorough resection, making tension-free inset challenging and potentially compromising its vascular supply due to compression. Rather than converting to free nipple grafting, which undoubtedly allows for greater tissue reduction but sacrifices vascular continuity, areolar sensitivity, and the potential of breast feeding, we prefer performing liposuction. As demonstrated by our study, PAL preserves the integrity of the NAC while effectively reducing its volume. Consequently, this procedure could also be beneficial in secondary breast reductions, particularly when the previously used technique and the position of the pedicle are unknown. By using liposuction, extensive undermining and wide excision of the pedicle can be reduced, thereby lowering the risk of NAC necrosis.

Consistent with previous studies utilizing liposuction for breast reduction, the proposed technique is most effective in patients with breast morphologies that predominantly consisted of adipose rather than dense glandular tissue [31,32,33,34]. Otherwise, liposuction will not evolve its benefits and contribute to an effective reduction and reshaping of the pedicle. Nevertheless, young or thin patients should not be precluded from this intervention [5]. Both women in our study aged ≤20 years with a BMI ≤ 25 kg/m^2^ successfully underwent complementary liposuction of at least 50 mL per side. In addition to clinical assessment, preoperative radiographic evaluation can help pre-selecting possible patients whose breasts have a predominant fat component over glandular tissue [16]. Yet, we prioritize intraoperative assessment of breast morphology.

When using hybrid methods, difficulties in assessing the right amount of volume infiltrated and aspirated are to be mentioned [35]. We always opt for symmetrical infiltration and liposuction of the pedicle when there is no obvious anisomastia present. Otherwise, it can be difficult to assess breast symmetry within the operation, especially after the installation of the tumescence solution. We gradually increased the amount of lipoaspirate obtained from the pedicle, reaching a maximum of 500 mL per side. In this regard, the risk of excessive emptying needs to be mentioned, most likely in elderly patients whose breasts have already undergone natural involution to adipose tissue [31]. If liposuction is too aggressively performed, a flattened shape (specifically in the upper pole) without projection might result. For this reason, we recommend a stepwise approach under repeated simulation of NAC rotation and assessment of breast size and shape to guide fat removal. In cases of preoperative anisomastia, careful intraoperative evaluation is even more essential. Larger breasts generally require both greater resection weights and higher lipoaspirate volumes, although there is no strict rule governing this relationship. The priority is to achieve uniform tumescence of the pedicle and adjacent areas to enable effective fat removal. Even after wound closure, if asymmetry or suboptimal breast volume persists, further liposuction can be performed to refine the shape and achieve the desired symmetry [20,35].

We did not use intraoperative Doppler sonography for mapping or dissecting of a definite vascular pedicle to the NAC as described by others [16]. Also, postoperative monitoring was performed exclusively through clinical examinations, assessing capillary refill and color, rather than using Doppler sonography. Based on our experience and the robust dual axial supply of the superomedial pedicle [22], we do not consider additional diagnostic equipment necessary for our surgical technique.

The major limitations of our study are its retrospective design and the small patient cohort. A prospective study, ideally with a matched control group, would have allowed for more robust data collection and stronger conclusions. Due to the small sample size statistical analysis was limited and did not allow for meaningful assessment of potential factors influencing liposuction volume, such as patient’s age or BMI. In addition, the lack of anthropometric data hinderedthe evaluation of whether certain body types (e.g., patients with small chest anteroposterior diameter) are more prone to developing massive breast ptosis with significant projection (as seen in Figure 4) than others.

Although patient satisfaction was subjectively high, a standardized patient survey during follow-up visits would have strengthened the assessment of long-term functional and aesthetic outcomes. This also applies for the evaluation of NAC sensibility. Sensory evaluation of the NAC is not routinely performed while patient counseling in our clinics. For this reason, we cannot provide a valuable assessment apart from subjective patient reporting during follow-up visits. All patients stated that NAC sensibility subjectively recovered after the operation. Yet, future studies should incorporate standardized pre- and postoperative NAC sensibility testing to better evaluate this aspect.

Furthermore, it would be valuable to assess whether complementary liposuction contributes to reduced postoperative pain levels. Since the tumescent solution used in PAL contains local anesthetics, it is plausible that this technique may provide enhanced pain control in the immediate postoperative period.

Ultimately, the study was limited to the practice of a single plastic surgeon at one institution. While this eliminates bias related to variations in surgical handling, experience, and intraoperative decision-making, it also restricts the generalizability of the findings. We would therefore appreciate it if other surgeons could further validate the technique’s reproducibility, reliability, and applicability and contribute to a multicenter study protocol.

## 5. Conclusions

Liposuction-assisted reduction of the superomedial pedicle may not be the treatment of choice for all patients, but we recommend considering the hybrid approach for women who opt for a significant reduction of ptotic and hypertrophic breasts that consist of an increased amount of adipose tissue. When intraoperative difficulties with pedicle insetting are encountered, liposuction can be performed until the desired volume and mobility are achieved. Based on our experience, complementary liposuction of the superomedial pedicle is a safe and effective technique that does not increase postoperative complications.

## Figures and Tables

**Table 1:** Representative demographic, intra-, and postoperative characteristics of the entire study cohort.

Pat. ID	Age (Years)	BMI (kg/m^2^)	Operation Time (min)	Resection Weight—Right (g)	Resection Weight— Left (g)	Liposuction Volume— Right (mL)	Liposuction Volume— Left (mL)	Postoperative Complications
1	44	37.8	146	1800	1570	450	300	Oil cyst
2	41	34.6	119	1300	1300	300	300	Oil cyst
3	43	32.7	150	1200	1200	200	200	No
4	18	22.1	115	700	790	80	100	No
5	67	36.2	116	1750	1450	500	400	No
6	36	27.8	116	850	850	80	80	No
7	40	24.7	132	1030	1050	150	200	Hematoma
8	36	31.5	111	1060	950	100	150	No
9	67	28.0	179	1550	1700	400	350	No
10	48	35.1	132	1500	1350	350	200	No
11	59	32.1	148	1450	1200	350	250	No
12	60	32.0	109	1100	1300	150	200	No
13	20	25.0	93	650	680	50	50	No
14	26	32.0	137	1610	1540	250	250	No
15	26	31.2	178	1700	1850	400	450	No

BMI: Body mass index; min: minutes; g: grams; mL: milliliters.

## Data Availability

All presented data are available upon reasonable request to the corresponding author I.A. Ederer. Reuse is only permitted after the agreement of all co-authors of this study.

## Supplementary Materials

The following supporting information can be downloaded at https://www.mdpi.com/article/10.3390/jcm14134475/s1: Video S1: Intraoperative video demonstrating PAL of the superomedial pedicle in Wise-pattern reduction mammoplasty.

## Abbreviations

The following abbreviations are used in this manuscript:

BMI	Body mass index
g	Grams
IMF	Inframammary fold
ISAPS	International Society of Aesthetic Plastic Surgery
mL	Milliliters
NAC	Nipple–areolar complex
PAL	Power-assisted liposuction
SD	Standard deviation

## References

[1] (2023). International Society of Aesthetic Plastic Surgery ISAPS Global Survey. https://www.isaps.org/media/rxnfqibn/isaps-global-survey_2023.pdf.

[2] Wise R.J. (1956). A Preliminary Report on a Method of Planning the Mammaplasty. Plast. Reconstr. Surg. (1946).

[3] McKissock P.K. (1972). Reduction Mammaplasty with a Vertical Dermal Flap. Plast. Reconstr. Surg..

[4] Georgiade N.G., Serafin D., Morris R., Georgiade G. (1979). Reduction Mammaplasty Utilizing an Inferior Pedicle Nipple-Areolar Flap. Ann. Plast. Surg..

[5] Lejour M. (1994). Vertical Mammaplasty and Liposuction of the Breast. Plast. Reconstr. Surg..

[6] Hall-Findlay E.J. (1999). A Simplified Vertical Reduction Mammaplasty: Shortening the Learning Curve. Plast. Reconstr. Surg..

[7] Orlando J.C., Guthrie R.H. (1975). The Superomedial Dermal Pedicle for Nipple Transposition. Br. J. Plast. Surg..

[8] Gray L.N. (1998). Liposuction Breast Reduction. Aesthetic Plast. Surg..

[9] Moskovitz M.J., Baxt S.A. (2004). Breast Reduction Using Liposuction Alone. Semin. Plast. Surg..

[10] Lejour M., Abboud M. (1990). Vertical Mammaplasty Without Inframammary Scar and With Breast Liposuction. Semin. Plast. Surg..

[11] Matarasso A. (2002). Suction Mammaplasty: The Use of Suction Lipectomy Alone to Reduce Large Breasts. Clin. Plast. Surg..

[12] Bustos S.S., Molinar V., Kuruoglu D., Cespedes-Gomez O., Sharaf B.A., Martinez-Jorge J., Manrique O.J., Tran N.V., Nguyen M.-D.T. (2021). Inferior Pedicle Breast Reduction and Long Nipple-to-Inframammary Fold Distance: How Long Is Safe?. J. Plast. Reconstr. Aesthetic Surg..

[13] Ederer I.A., Spennato S., Kueenzlen L., Kuehn S., Rothenberger J., Rieger U.M. (2021). Overcoming the Limits of Traditional Breast Reduction -Inferior Pedicle Approach for Macromastia with Sternal Notch-to-Nipple Distance of up to 57 Cm. J. Plast. Reconstr. Aesthetic Surg..

[14] Talwar A.A., Copeland-Halperin L.R., Walsh L.R., Christopher A.N., Cunning J., Broach R.B., Baratta M.D., Copeland M., Shankaran V., Butler P.D. (2023). Outcomes of Extended Pedicle Technique vs Free Nipple Graft Reduction Mammoplasty for Patients With Gigantomastia. Aesthetic Surg. J..

[15] Bonomi F., Harder Y., Treglia G., De Monti M., Parodi C. (2024). Is Free Nipple Grafting Necessary in Patients Undergoing Reduction Mammoplasty for Gigantomastia? A Systematic Review and Meta-Analysis. J. Plast. Reconstr. Aesthetic Surg..

[16] Sağır M., Güven E. (2024). Expanding Usage of Superior Pedicled Techniques with Reducing Resistance in the Pedicle by Lipoaspiration. Aesthetic Plast. Surg..

[17] Hall-Findlay E.J. (2002). Vertical Breast Reduction with a Medially-Based Pedicle. Aesthetic Surg. J..

[18] Akyurek M. (2011). Contouring the Inferior Pole of the Breast in Vertical Mammaplasty: Suction-Assisted Lipectomy versus Direct Defatting. Plast. Reconstr. Surg..

[19] Price M.F., Massey B., Rumbolo P.M., Paletta C.E. (2001). Liposuction as an Adjunct Procedure in Reduction Mammaplasty. Ann. Plast. Surg..

[20] Abboud M.H., Dibo S.A. (2016). Power-Assisted Liposuction Mammaplasty (PALM): A New Technique for Breast Reduction. Aesthetic Surg. J..

[21] Yang Z., Li F., Han X., Cai L., Yin B. (2020). Application of Liposuction Technique Assisted Superomedial Pedicle with Vertical Incision in Reduction Mammaplasty. Zhongguo Xiu Fu Chong Jian Wai Ke Za Zhi.

[22] Hall-Findlay E.J. (2024). Breast Reduction: What I Have Changed over the Years. Australas. J. Plast. Surg..

[23] Brown R.H., Siy R., Khan K., Izaddoost S. (2015). The Superomedial Pedicle Wise-Pattern Breast Reduction: Reproducible, Reliable, and Resilient. Semin. Plast. Surg..

[24] Hall-Findlay E.J., Shestak K.C. (2015). Breast Reduction. Plast. Reconstr. Surg..

[25] Huang S.H., Wu S.H., Chang K.P., Wang W.H., Lai C.H., Sun I.F., Lin S.D., Lai C.S. (2009). Contour Refinements of Free Flaps for Optimal Outcome in Oral Reconstruction: Combination of Modified Liposuction Technique and w-Plasty in One-Stage Procedure. J. Craniomaxillofac. Surg..

[26] Brorson H., Svensson H., Norrgren K., Thorsson O. (1998). Liposuction Reduces Arm Lymphedema without Significantly Altering the Already Impaired Lymph Transport. Lymphology.

[27] Bertheuil N., Chaput B., De Runz A., Girard P., Carloni R., Watier E. (2017). The Lipo-Body Lift: A New Circumferential Body-Contouring Technique Useful after Bariatric Surgery. Plast. Reconstr. Surg..

[28] Brorson H. (2016). Liposuction in Lymphedema Treatment. J. Reconstr. Microsurg..

[29] Fan S., Zhang H., Li Q., Tian T., Chen W., Pan G., Ahn M.H.Y., Sun S., Wu B., Li J. (2018). The Use of a Honeycomb Technique Combined with Ultrasonic Aspirators and Indocyanine Green Fluorescence Angiography for a Superthin Anterolateral Thigh Flap: A Pilot Study. Plast. Reconstr. Surg..

[30] Bertheuil N., Chaput B., Berger-Müller S., Ménard C., Mourcin F., Watier E., Grolleau J.L., Garrido I., Tarte K., Sensébé L. (2016). Liposuction Preserves the Morphological Integrity of the Microvascular Network: Flow Cytometry and Confocal Microscopy Evidence in a Controlled Study. Aesthetic Surg. J..

[31] Auersvald A., Botti C., Botti G., Pascali M. (2022). Liporeduction: A Faster and Safer Breast Remodeling Technique. Plast. Reconstr. Surg..

[32] Kadhum M., Symonette C., Khan W., Javed M.U. (2024). Liposuction-Only Breast Reduction: A Systematic Review of Outcomes. Aesthetic Plast. Surg..

[33] Habbema L. (2009). Breast Reduction Using Liposuction with Tumescent Local Anesthesia and Powered Cannulas. Dermatol. Surg..

[34] Jakubietz R.G., Jakubietz D.F., Gruenert J.G., Schmidt K., Meffert R.H., Jakubietz M.G. (2011). Breast Reduction by Liposuction in Females. Aesthetic Plast. Surg..

[35] Hamdi M. (2016). Commentary on: Power-Assisted Liposuction Mammaplasty (PALM): A New Technique for Breast Reduction. Aesthetic Surg. J..

